# Sonographic examination of the median nerve in dialysis patients and after renal transplantation

**DOI:** 10.1002/brb3.406

**Published:** 2015-11-14

**Authors:** Anne Elisabeth Carolus, Peter Schenker, Thomas Dombert, Johann Fontana, Richard Viebahn, Kirsten Schmieder, Christopher Brenke

**Affiliations:** ^1^ Department of NeurosurgeryUniversity Hospital Knappschaftskrankenhaus BochumRuhr‐University BochumBochumGermany; ^2^ Department of General, Visceral and Transplant SurgeryUniversity Hospital Knappschaftskrankenhaus BochumRuhr‐University BochumBochumGermany; ^3^ Center for Peripheral Nerve SurgeryDossenheim‐HeidelbergGermany

**Keywords:** Carpal tunnel syndrome, cross‐sectional area, dialysis, median nerve, renal transplantation, ultrasonography

## Abstract

**Objective:**

Patients with renal insufficiency are predisposed to develop CTS (carpal tunnel syndrome). In particular, long‐term dialysis seems to contribute to changes in median nerve texture which lead to an increased risk for CTS. The current study was designed to evaluate if these structural changes can be detected by HRS (high‐resolution sonography). Additionally, the current study aimed to determine if changes are reversible after termination of dialysis.

**Methods:**

Fifty patients (98 hands) were included in the study. The study population was subdivided into three groups: patients without any history of renal disease (H, *n* = 20), patients with long‐term dialysis (D, *n* = 10), and patients after renal transplantation (TX,* n* = 20). None of the patients had any clinical symptoms for a median nerve compression syndrome. The CSA (cross‐sectional area) of the median nerve was evaluated both 12 cm proximally of the carpal tunnel inlet and directly at the carpal tunnel inlet. The ratio of those two values, the WFR (wrist forearm ratio), was calculated and analyzed.

**Results:**

The CSA demonstrated significantly higher values in dialysis (D) and transplanted (TX) patients compared to the healthy (H) control group (*P* < 0.001). No significant differences were detectable between the D and TX groups. Specifically, there was no significant difference in the WFR.

**Conclusion:**

Patients with chronic renal disease demonstrate significantly higher CSA values compared to their healthy counterparts. Termination of dialysis does not seem to reverse these morphological changes.

## Introduction

CTS (carpal tunnel syndrome) in patients with renal insufficiency and long‐term dialysis is a common neurological complication with up to 32% prevalence (Kessler et al. 1992; Ibrahim et al. 2012; Baumgaertel et al. 2014). The exact pathomechanisms are not yet definitely identified. The preferred theory is that dialysis‐associated amyloidosis with accumulation of *β*2‐microglobulin in the blood leads to deposits in the bone and joint tissue. This could result in nerve affection in the carpal tunnel (Kessler et al. 1992; Hoshino et al. 2014; Nishi 2014). Another consideration is that an increased vulnerability of the median nerve in cases of pre‐existing polyneuropathy could predispose for entrapment neuropathy (Kwon et al. 2011). In addition to the case history and clinical examination, electrodiagnostic testing is currently considered the gold standard for confirmation of a clinical diagnosis of CTS (Fowler et al. 2014). However, HRS (high‐resolution sonography) gains in importance due to several advantages, such as it is cost‐effective, noninvasive, and easy to use. Recent analyses prove a good sensitivity and specificity for HRS in the diagnosis of CTS (Wong et al. 2002; Roll et al. 2011; Tai et al. 2012; Fowler et al. 2014). In addition, it can be regarded as a sectional imaging technique, which can supply morphological information that NCS (Nerve conduction study) cannot provide (Pazzaglia and Padua 2011). The nerve CSA (cross‐sectional area) at the inlet of the carpal tunnel and WFR (wrist forearm ratio), respectively, are considered the most significant parameters (Wong et al. 2002; Pazzaglia and Padua 2011; Tai et al. 2012). CSA measurements can indicate a swelling of the nerve provoked by chronic compression. There are authors who conclude that the degree of median nerve swelling is not only an indicator for CTS, but also reflects the severity of clinical symptoms (El Miedany et al. 2004; Karadağ et al. 2010).

The aim of our study was to screen a group of individuals with the predisposition factor “renal disease” for texture changes of the median nerve. To the best of our knowledge, this has not been carried out so far. We examined patients in two different states of renal disease: the state of terminal dialysis dependence and the postdialysis state after renal transplantation. CSA and then the WFR (wrist forearm ratio) are used as parameters. The main question is, can a pathological enlargement of CSA be seen in patients who undergo dialysis frequently even in a subclinical state without symptoms for CTS? Furthermore, it is unclear whether renal transplantation leads to a normalization of pathological CSA.

## Materials and Methods

This is a prospective study involving a total of 50 participants and 98 single hands. The study population was subdivided into three groups: *n* = 20 without any history of renal disease (H; 10 woman, 10 men, age 45.8 ± 14.3 years, range 26–79 years), *n* = 10 with long‐term dialysis for at least 6 months (D; 6 woman, 4 men, age 55.6 ± 15.2 years, range 32–74 years), and *n* = 20 after renal transplantation more than 6 months earlier (TX; 7 women, 13 men, age 56.25 ± 9.79 years, range 34–76 years). All transplanted patients had a stable function of the kidney graft with an average serum creatinine of 1.6 ± 0.3 mg/dL and a mean glomerular filtration rate of 54.1 ± 7.3 mL/min. Participants with other risk factors for CTS (i.e., diabetes mellitus, rheumatoid arthritis, pathology in the cervical spine, preliminary trauma, or surgery in wrist region) were excluded. None of the female participants was pregnant. Two hands with an anatomical variant as a bifid median nerve were also excluded.

None of the patients had signs or symptoms for CTS defined according to the criteria of the American Academy of Neurology practice parameters (Subcommittee of the American Academy of Neurology, 1993). A questionnaire was used to exclude history of CTS and other risk factors. Furthermore, to assess the degree of stress to the wrists, whether participants were right‐ or left‐handed, their professions, leisure activities, and everyday life activities were recorded.

**Figure 1:**
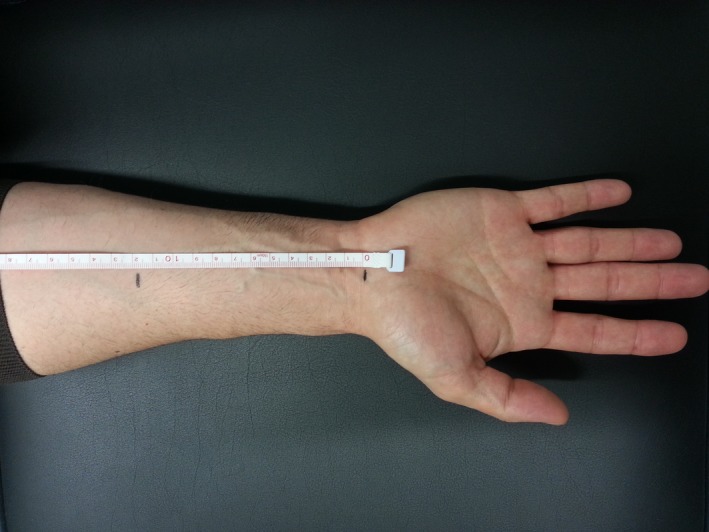
The two forearm levels where median nerve cross‐sectional area are measured by high‐resolution sonography: 12 cm proximally from wrist (R/L12) and directly above the wrist crease (R/L0).

**Figure 2:**
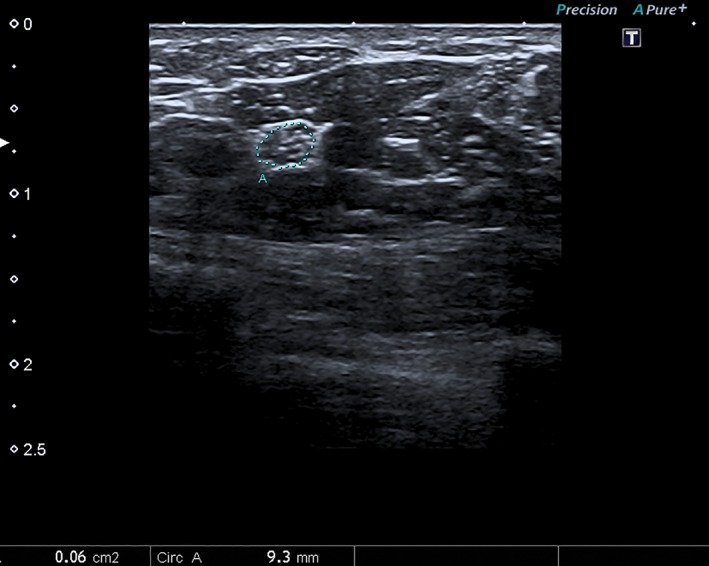
Median nerve cross‐sectional area (A) measured in the 12 cm forearm level using continuous tracing method.

**Figure 3:**
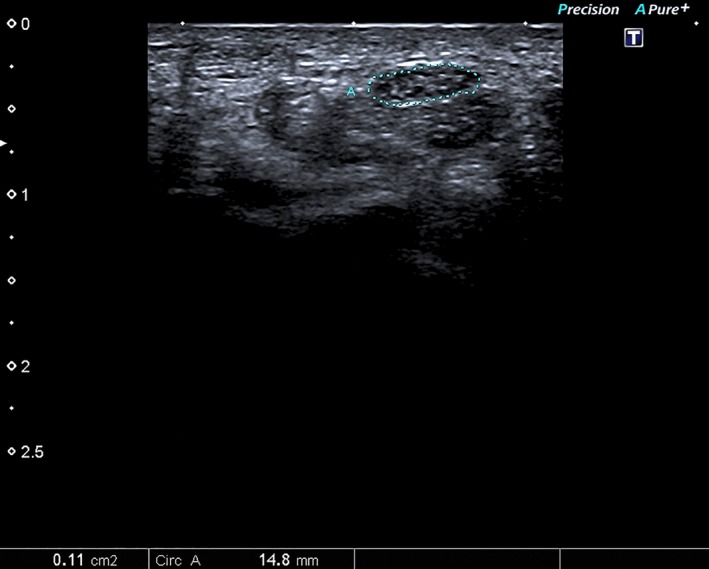
Median nerve cross‐sectional area (A) measured at carpal tunnel inlet using continuous tracing method.

All patients underwent clinical examination including an inspection with the focus on muscle atrophy, skin coloration, and swelling; typical provocation tests; an examination of the muscles innervated by the median nerve; and an examination of sensory abilities. The wrist circumference of each hand was measured by taking a tape measure around the wrist bone. The sonography was performed using a Toshiba Aplio 500 machine (TOSHIBA Medical Systems GmbH, Neuss, Germany) equipped with a high‐frequency 14 MHz linear array transducer. All examinations were performed by the same sonographer following a standardized protocol of scanning parameters (e.g., frequency, depth). Patients were examined in a sitting position, hands lying with palm up and fingers semi‐extended. The CSA of the median nerve was measured in two different levels: 12 cm proximally of wrist in the forearm region (R12/L12) and directly above the rasceta (R0/L0) (Fig. 1). We used the continuous tracing method of the nerve circumference (Figs. 2, 3). Measurement was taken at the inner border of the hyperechoic rim of the nerve. To define the cutoff values of CSA measured at the proximal inlet of CTS, a detailed PubMed Database research was carried out. We included the study of El Miedany et al. (2004) and defined the values as following: 10.0–13.0 mm^2^ for mild, 13.0–15.0 mm^2^ for moderate, and >15.0 mm^2^ for severe nerve compression.

The local ethics board of the Faculty of Medicine, Ruhr‐University of Bochum, approved the study. All participants gave their written consent. Patients were recruited from the department of transplantation, University Hospital Knappschaftskrankenhaus Bochum between September and December 2014. Statistical analyses were performed using SPSS version 22.0 (SPSS Inc., Chicago, IL). The descriptive data were expressed as a mean value. A one‐way ANOVA (analysis of variance) and post hoc test were used to detect differences of CSA in different forearm levels. The chi‐square test was used to compare categorical variables. Intergroup frequencies were compared using the Fisher's exact test for categorical variables and the Student's *t*‐test for continuous variables. *P*s < 0.05 were considered to be statistically significant.

## Results

The baseline characteristics of the participants are listed in Table 1. There was an equal distribution of gender in the H group. In the D and TX group, we had a slight gender imbalance in favor of the male participants. Concerning age, there was a significant difference between the H and the D group (*P* = 0.04) with younger participants in H, but no significant difference between the H and the TX group (*P* = 0.16). There was no significant difference between age in the D and the TX group (*P* = 1.0). In most cases the participants were right‐handed, only two people of the TX group showed left‐hand dominance. Since the CSA values did not show a significant difference between the dominant and not‐dominant side, we regarded each hand as an independent variable. For the D group, there was a mean period of dialysis treatment of 72.5 ± 58.7 months with a range from 6 to 168 months. For the TX group, the mean time since termination of dialysis treatment was 57.9 ± 40.2 months with a range from 6 to 155 months.

**Table 1:** Patient characteristics

	Healthy control group (H)	Dialysis group (D)	Renal transplantation group (TX)
Patients	20	10	20
Hands	40	20	40
Dominant hand (right:left)	20/0	10/0	18/2
Gender (male/female)	10/10	6/4	13/7
Age (years)	45.80 ± 14.36	55.60 ± 15.28	56.25 ± 9.79
Time since start of dialysis/termination of dialysis (months)	0	72.5 ± 58.70	57.95 ± 40.23

Values are given as mean ± SD or *n*.

Table 2 shows the mean values of the anatomical measurements and sonographic parameters. The mean wrist circumference was 16.75 ± 1.25 cm for the right hand and 16.56 ± 1.14 cm for the left hand in the H group, 17.50 ± 1.52 cm for the right hand and 17.50 ± 1.50 cm for the left hand in the D group, and 17.03 ± 1.21 cm for the right hand and 16.19 ± 3.31 cm for the left hand in the TX group, without statistical significant differences. The H group demonstrated CSA values with a mean of 4.2 ± 1.4 mm² at the R12 (right hand/forearm) level and a mean of 4.9 ± 1.12 mm² at the L12 (left hand/forearm) level. There was a mean of 7.6 ± 1.5 mm² at the R0 (right hand/carpal tunnel inlet) level and a mean of 7.56 ± 1.8 mm² at the L0 (left hand/carpal tunnel inlet) level. The D group demonstrated CSA values with a mean of 6.0 ± 1.6 mm² at the R12 level and a mean of 7.4 ± 1.2 mm² at the L12 level. There was a mean of 10.3 ± 1.9 mm² at the R0 level and a mean of 9.7 ± 1.2 mm² at the L0 level. The TX group demonstrated CSA values with a mean of 6.4 ± 2.1 mm² at the R12 level and a mean of 5.6 ± 1.5 mm² at the L12 level. There was a mean of 10.5 ± 2.1 mm² at the R0 carpal tunnel inlet level and a mean of 9.8 ± 2.1 mm² at the L0 carpal tunnel inlet level. The mean of WFR in the H group was 1.95 ± 0.81 for the right and 1.91 ± 0.90 for the left hand. The mean of WFR in the D group was 1.86 ± 0.66 for the right and 1.33 ± 0.42 for the left hand. The mean of WFR in the TX group was 1.88 ± 0.73 for the right and 1.84 ± 0.58 for the left hand. The multiple paired comparison of the CSA is depicted in Table 3. The calculation there includes both the right and the left hand. It shows significantly higher CSA values in the D group compared to the H group in both levels R/L12 and R/L0 (*P* < 0.001 and *P* < 0.001). The difference in CSA between the TX group and the H group was significant as well (*P* < 0.001 and *P* < 0.001). No significant differences were detectable between the D and TX groups (*P* = 0.242 and *P* = 0.100). According to the findings listed above, there was no significant difference in the WFR in all paired comparisons.

**Table 2:** Anatomic and sonographic measurements

	Healthy control group (H)	Dialysis group (D)	Renal transplantation group (TX)
CW [cm]			
R	16.75 ± 1.25	17.50 ± 1.52	17.03 ± 1.21
L	16.56 ± 1.14	17.50 ± 1.50	16.19 ± 3.31
CSA [mm²]			
R12	4.2 ± 1.4	6.0 ± 1.6	6.4 ± 2.1
R0	7.6 ± 1.5	10.3 ± 1.9	10.5 ± 2.1
L12	4.9 ± 1.12	7.4 ± 1.2	5.6 ± 1.5
L0	7.56 ± 1.8	9.7 ± 1.2	9.8 ± 2.1
WFR			
R	1.956 ± 0.814	1.863 ± 0.665	1.886 ± 0.730
L	1.914 ± 0.903	1.334 ± 0.261	1.847 ± 0.582

Values are given as mean ± SD. CSA, cross‐sectional area; WFR, wrist and forearm ratio; CW, circumference of wrist; R, right; L, left.

**Table 3:** Multiple paired comparison. CSA, WFR, and CW as dependent variables

Study population	Study population	Difference in mean CSA [mm²] R/L12*	*P*‐value	Difference in mean CSA [mm²] R/L0*	*P*‐value	Difference in WFR	*P*‐value	Difference in mean CW [cm]	*P*‐value
TX	H	1.3833	<0.001	2.5675	<0.001	0.0701	1.000	0.288	1.000
TX	D	−0.7526	0.242	0.1316	1.000	0.2666	0.500	−0.461	1.000
D	H	2.1359	<0.001	2.4359	<0.001	0.3367	0.286	−0.750	0.736

TX, renal transplant group; H, healthy group; D, dialysis group; CSA, cross‐sectional area; WFR, wrist and forearm ratio; CW, circumference of wrist; *Calculation includes right and left hand.

## Discussion

It is not a new finding that subclinical median nerve compression is common in the healthy population (Atroshi et al. 1999) and occurs with even higher prevalence in patients with certain risk factors. Our study selectively screened a population with one predisposition factor. We attempted to exclude all concomitant diseases, which are known to be associated with CTS. The investigation demonstrates a significantly larger CSA for median nerve in wrists of participants who underwent long‐term dialysis compared to their healthy counterparts. The precise CSA values were found around the cutoff point for mild CTS (El Miedany et al. 2004; Karadağ et al. 2010; Tai et al. 2012). Thus, the sonographic results confirm the high prevalence of CTS in those individuals. But although dialysis patients develop CSA enlargement with the most probability, it is unclear which factors lead to subclinical nerve abnormalities becoming clinically apparent CTS. This becomes even more obvious if we consider the duration of treatment, which shows a wide range from a minimum of 6 months up to 168 months in our study population. The investigated participants who underwent renal transplantation showed CSA values of mild CTS equally to the dialysis group. Furthermore, CSA proved to be independent of the dialysis‐free period ranging from 6 months as minimum up to 155 months in our study. This is corresponding to the first observation, namely that the fact but not the duration of dialysis treatment seems to have an influence of CSA enlargement. The enlargement of CSA seems to be irreversible or at least nonreversible for a long time after normalization of renal‐ and dialysis‐related metabolic processes.

Our investigation has to be considered as a preliminary study with several clear limitations.

First, diabetes mellitus is a frequent concomitant disease of dialysis patients. Since we defined it as an exclusion criteria, group D comprises only *n* = 10 participants which is a small number for statistical analysis.

Although we included individuals in two different states of renal disease, our study was a one‐time investigation and we did not pursue the clinical and sonographic course of each participant. Therefore, this nonsequential character of the study does not allow us to make conclusions of causal relationships.

As already mentioned, there is an increasing interest on HRS as a tool in the peripheral nerve field, particularly in the diagnostic of CTS. But it is uncontroversial that it remains an investigator‐dependent instrument. Well‐experienced examiners use HRS with a high intrarater, interrater, and interequipment specificity (Karadağ et al. 2010; Tai et al. 2012; Boehm et al. 2014). Nevertheless, it is a technique in an ongoing state of development. Although reference values for CSA in different nerve segments have recently been established, respectively, defined more in detail (Boehm et al. 2014), the conventional conduction study has still to be considered as the proven gold standard (Fowler et al. 2014) for diagnosis of peripheral nerve entrapment syndromes such as CTS.

With regard to that it is important to emphasize that our study does not provide information of HRS advantages or disadvantages. There is a need for a better understanding of the underlying pathophysiology of CTS in renal patients and an imaging tool as HRS could provide that.

## Conclusion

High‐resolution sonography verifies the expected high prevalence of morphological nerve abnormalities in individuals with renal insufficiency in the dialysis and postdialysis state. Thus, it is able to reveal subclinical nerve compression, but it does not seem to be a diagnostic predictor for clinically relevant CTS. Due to the current results, consequences for the clinical practice, particularly an early surgical management, do not seem to be justified. Follow‐up studies comprising long‐term examinations, an enlarged collective of patients, and electrophysiology as a complementary diagnostic tool are necessary to clarify the clinical significance of HRS findings.

## Conflict of Interest

None declared.
